# Honokiol Inhibits Atrial Metabolic Remodeling in Atrial Fibrillation Through Sirt3 Pathway

**DOI:** 10.3389/fphar.2022.813272

**Published:** 2022-03-17

**Authors:** Guang Zhong Liu, Wei Xu, Yan Xiang Zang, Qi Lou, Peng Zhou Hang, Qiang Gao, Hang Shi, Qi Yun Liu, Hong Wang, Xin Sun, Cheng Liu, Peng Zhang, Hua Dong Liu, Shao Hong Dong

**Affiliations:** ^1^ Department of Cardiology, Shenzhen Cardiovascular Minimally Invasive Medical Engineering Technology Research and Development Center, Shenzhen People’s Hospital, Shenzhen, China; ^2^ Shenzhen People’s Hospital, The Second Clinical Medical College, Jinan University; The First Affiliated Hospital, Southern University of Science and Technology, Shenzhen, China; ^3^ Department of Cardiology, the First Affiliated Hospital, Harbin Medical University, Harbin, China; ^4^ Department of Pharmacy, Clinical Medical College, Yangzhou University, Northern Jiangsu People's Hospital, Yangzhou, China

**Keywords:** Honokiol, atrial fibrillation, metabolism remodeling, sirt3, acetylation

## Abstract

**Background and Purpose:** Atrial metabolic remodeling plays a critical role in the pathogenesis of atrial fibrillation (AF). Sirtuin3 (Sirt3) plays an important role in energy homeostasis. However, the effect of Sirt3 agonist Honokiol (HL) on AF is unclear. Therefore, the aim of this study is to determine the effect of HL on atrial metabolic remodeling in AF and to explore possible mechanisms.

**Experimental Approach:** irt3 and glycogen deposition in left atria of AF patients were examined. Twenty-one rabbits were divided into sham, P (pacing for 3 weeks), P + H treatment (honokiol injected with pacing for 3 weeks). The HL-1 cells were subjected to rapid pacing at 5 Hz for 24 h, in the presence or absence of HL and overexpression or siRNA of Sirt3 by transfection. Metabolic factors, circulating metabolites, atrial electrophysiology, ATP level, and glycogens deposition were detected. Acetylated protein and activity of its enzymes were detected.

**Key Results:** Sirt3 was significantly down-regulated in AF patients and rabbit/HL-1cell model, resulting in the abnormal expression of its downstream metabolic key factors, which were significantly restored by HL. Meanwhile, AF induced an increase of the acetylation level in long-chain acyl-CoA dehydrogenase (LCAD), AceCS2 and GDH, following decreasing of activity of it enzymes, resulting in abnormal alterations of metabolites and reducing of ATP, which was inhibited by HL. The Sirt3 could regulate acetylated modification of key metabolic enzymes, and the increase of Sirt3 rescued AF induced atrial metabolic remodeling.

**Conclusion and Implications:** HL inhibited atrial metabolic remodeling in AF *via* the Sirt3 pathway. The present study may provide a novel therapeutical strategy for AF.

## Introduction

Atrial fibrillation (AF) is the most common sustained arrhythmia in clinical practice and may eventually be associated with morbidity and mortality. Recently, metabolomic and proteomic studies in humans and experimental AF have reported changes in the expression of molecules involved in metabolic pathways, indicating a role for metabolic alterations in the pathogenesis of AF (Mayr et al., 2008; Tu et al., 2014b). However, it is not clear that the precise mechanism underlying the impact of atrial metabolic remodeling on AF persistence.

Sirtuins are a family of nicotinamide adenine dinucleotide- (NAD+) dependent histone deacetylases and thus their function is intrinsically linked to cellular metabolism (Chang and Guarente, 2014; van de Ven et al., 2017). Sirt3 is a deacetylase that regulates the activity of the key enzymes *via* deacetylation, resulting in regulating mitochondrial energy metabolism (Lombard et al., 2007; Rardin et al., 2013). Sirt3-mediated deacetylation modifies and activates long-chain acyl-CoA dehydrogenase (LCAD) (Hirschey et al., 2010), and regulates other enzymes of fatty acid oxidation, such as medium chain-specific acyl-CoA dehydrogenase (ACADM) (Yang et al., 2016). In addition, Sirt3 deacetylate essential enzymes of acetyl-CoA synthetase 2(AceCS2) (Schwer et al., 2006)and isocitrate dehydrogenase 2(IDH2) (Yu et al., 2012) are involved in the tricarboxylic acid (TCA) cycle, and the glutamate dehydrogenase (GDH) in the urea cycle of metabolism (Kim et al., 2012). Our previous studies found that AF induced an expressed imbalance of the metabolic factor involved in fatty acid and glucose oxidation, which is involved in the down-regulation of PPAR-α/sirtuin1/PPAR co-activator α (PGC-1α) pathway (Liu et al., 2016). However, the identity and role of Sirt3 under AF remains unknown.

Honokiol (HL) is a small molecular weight natural compound derived from Magnolia grandiflora, which is used as a traditional Chinese herb. A report has shown that HL could ameliorate cardiac hypertrophy by binding to Sirt3, activating it and increasing Sirt3 levels and its enzymatic activity (Pillai et al., 2015). Furthermore, activation of Sirt3 by Honokiol increased ATP production as well as reduced ROS and lipid peroxidation by improving fatty acid oxidation resulting in the inhibition of acute kidney injury induced by cisplatin (Li et al., 2020). However, the effects of HL on the atrial metabolic remodeling associated with AF are not completely understood.

Therefore, our study was designed to investigate whether HL prevented atrial metabolic remodeling in AF through regulating acetylated modification of key metabolic enzyme by the Sirt3 pathway.

## Materials and Methods

### Ethics Statement

The use of animals and all procedures were in accordance with the Guide for the Care and Use of Laboratory Animals (NIH Publication 2011; eighth edition) and were approved by the Animal Care and Use Committee of the Harbin Medical University. All animals received a standard laboratory diet and filtered water ad libitum. They were housed in individual cages in a temperature-controlled room at 23–25°C under a 12 h light-dark cycle. All animal procedures were conducted in accordance with the ARRIVE guidelines (Kilkenny et al., 2010; McGrath et al., 2010).

### Clinic Patient Selection

The study abided by the principles that govern the use of human tissues outlined in the Declaration of Helsinki. All patients recruited into the study provided informed consent for their samples to be used. Left atrial appendages were obtained as surgical specimens from patients undergoing cardiac surgery for mitral valve replacement following established procedures approved by the local Ethics Committee (application approval numbers: 201551). Samples were collected from patients with sinus rhythm (SR, n = 7, without history of AF) and permanent AF (n = 7, documented arrhythmia for >6 months before surgery). Patients were excluded from the study if they had other cardiac diseases, such as severe congestive heart failure, or serious systemic diseases such as thyroid disease, impaired glucose tolerance or diabetes mellitus. The specimens were immediately fixed in 4% paraformaldehyde for 48 h at 4°C and stored at −80°C. The clinical subject characteristics were shown in Table 1.

**TABLE 1:** The clinical subject characteristics (mean ± SEM).

	SR n = 7	PAF n = 7	*p* Value
Age(years)	49.29 ± 4.05	57.86 ± 2.82	0.108
BW(kg)	70.29 ± 6.90	66.29 ± 5.49	0.653
BMI(kg/㎡)	24.51 ± 1.68	23.78 ± 1.55	0.753
EF (%)	61.00 ± 2.36 (n = 5)	63.80 ± 4.14 (n = 5)	0.573
Medication spirolactone	7/7	7/7	
furosemide	7/7	7/7	
AngiotensinⅡreceptor blocker (ARB)	1/6	0/6	
NYHA class (II/III)	5/2	4/3	

The data were expressed as mean ± SEM. SR, sinus rhythm; PAF, permanent atrial fibrillation; BW, body weight; BMI, body mass index; EF, ejection fraction; NYHA, new york heart association classification.

### Rabbit AF Model

The twenty-one New Zealand white rabbits (male, 2.5–3.0 kg) were provided by the Experimental Animal Center of the First Affiliated Hospital of Harbin Medical University and were randomly chosen in accordance with its weight divided into three groups: sham surgery group (sham, n = 7) with sutured electrodes and no pacing; Pacing group (P, n = 7) with an AF model induced by rapid right atrial pacing for 3 week at 600 beats/min; Honokiol treatment group (P + H group, n = 7) with Honokiol intraperitoneally injected (MedChemExpress,Cat.No HY-N0003) at a dose of 5 mg kg^−1^ day^−1^ for 21 days (Chiang et al., 2009; Sulakhiya et al., 2014) and pacing the right atria for 3 weeks. The AF model was established according to our previous studies (Li et al., 2007; Liu et al., 2013). All rabbits were allowed to recover for 1 week after the surgery. The rabbits were anaesthetized with ketamine (30–35 mg/kg) and xylazine (sigma; 5 mg/kg i. m.). All blood samples were collected after an overnight fast and serum was separated and stored at −80°C prior to analysis.

### Cell Culture and Transfection

The HL-1 cells were cultured in flasks in Claycomb medium (Sigma-Aldrich, United States) supplemented with10% foetal calf serum, 1% penicillin/streptomycin, and Norepinephrine (0.1 mM,SigmaAldrich, United States), and l-glutamine (2mM, Sigma-Aldrich,United States) at 37°C in 5% CO_2_. HL-1 cells were cultured in well plates and subjected to tachypacing by the stimulator (YC-2 stimulator) as described according to previous studies (Yang et al., 2005; Brundel et al., 2006). Cells (≥1×10^6^ myocytes) were stimulated at the parameter (5 Hz with square pulses of 5 ms duration, pulse voltage of 1.5 V/cm). HL-1 cells were transfected with 100 μM Sirt3 siRNA after 24 h plating by using Lipofectamine 2000 (Invitrogen) in OptiMem (Gibco) media. Cells were returned to growth media for 6 h after transfection. To specifically overexpress Sirt3, plasmid (OriGene Technologies, Inc.) was constructed. HL-1cells were transfected with 2 μg plasmid after 24 h plating.

### Electrophysiological Stud

The atrial electrophysiology detection was performed as described in previous study (Zhao et al., 2010). 8 basic stimuli (S1) were followed by a premature extra stimulus (S2), and the S1S1 cycle were both 150 and 200 ms basic cycle lengths (BCLs). The interval of S1-S2 was decreased by 10 ms and then decreased in 2 ms steps until S2 failed to capture the depolarization which was defined as the AERP value. The AERP value was tested 2 times at both BCLs: 200 ms (AERP_200_) and 150 ms (AERP_150_) to obtain the mean value of the 2 AERPs. AF vulnerability was determined as the percentage of AF and the atrial arrhythmia recorded with an intracardiac electrode sustained for ≥1 s induced by a train of 10 Hz, 2 ms stimuli to the right atrium at each interval of 2 min.

### Real-Time RT-PCR

Total RNA was extracted with reagent (Axygen, United States). The quantitative real-time reverse transcriptase-polymerase chain reaction (RT-PCR) was used according to a previously described procedure (Zhao et al., 2017). The real-time PCR was performed on the Applied Bio-system (Foster City, CA, United States). The primers of related genes used in the study are listed in Table 2.

**TABLE 2:** Primers for real-time PCR.

Gene name	Primer sequences	Product size (bp)
Sirt3 forward primer	TGC​CAG​AGG​GTG​GTG​GTC​AT	169
Sirt3 reverse primer	GAC​CTC​CAT​CAG​CCC​CAA​A	
LCAD forward primer	GGG​TGG​TTA​AGT​GAT​GTT​GT	127
LCAD reverse primer	GTA​GCT​TCT​GTC​CCT​TGA​TA	
LDHa forward primer	ACG​GCA​GCA​AGA​GGG​AGA​AA	313
LDHa reverse primer	GTA​ACG​GAA​GCG​GGC​TGA​AT	
AceCS2 forward primer	GCG​TTT​GCC​TTC​GTC​GTG​AT	101
AceCS2 reverse primer	CGG​CGT​ATT​TGG​CGA​TTT​T	
PGC-1αforward primer	TGA​TGA​CAG​CGA​AGA​TGA​AAG​TG	133
PGC-1αreverse primer	TTT​GGG​TGG​TGA​CAC​GGA​AT	
NDUFA9 forward primer	GCA​GAC​GCC​CGA​GGG​AAA​AC	114
NDUFA9 reverse primer	CAA​GGG​GTA​TGG​GAG​GAA​GG	
GLUT1 forward primer	GGC​AGA​TGA​TGC​GGG​AGA​AG	235
GLUT1 reverse primer	ACG​AAC​AGC​GAT​ACG​ACG​GT	
PDK4 forward primer	CTT​CAG​TTA​CAC​ATA​CTC​CAC​CGC	86
PDK4 reverse primer	GTA​ACC​CGT​AAC​CGA​AAC​CAG	
PDH forward primer	GCC​AAT​CAT​AAA​AGA​CGC​TG	150
PDH reverse primer	ATG​CCA​AAC​ATC​CCC​AAG​T	
GDH forward primer	CTG​GAT​GAA​GCG​GGA​AAG​GG	288
GDH reverse primer	GGG​CGG​CAC​GGA​GAA​GTA​GA	
CROT forward primer	GGC​TTC​GAC​CGT​CAC​CTT​CT	216
CROT reverse primer	CCT​GTC​GTC​TCG​GAT​GTG​GT	
SDHa forward primer	GGG​GAG​TGT​CGT​GGT​GTT​ATC	109
SDHa reverse primer	TGA​AGT​AAG​TGC​GCC​CAT​AGC	
β-actin forward primer	AGA​TCG​TGC​GGG​ACA​TCA​AG	182
β-actin reverse primer	CAG​GAA​GGA​GGG​CTG​GAA​GA	

### Western Blotting

The western blotting procedures were performed as described in a previous study (Anfuso et al., 2017). The 30–50 μg proteins were transferred to a polyvinylidene fluoride membrane. Membranes were blocked by 5% non-fat milk for 1 h. Then, the membranes were incubated overnight at 4°C with primary antibodies against Sirt3 (1:500, Abcam, 28kD), AceCS2 (1:500, Abclonal, 75kD), LCAD (1:500,Abclonal, 47kD), GDH (1:500,Abcam, 89kD), and β-actin (1:500, sigma, 42kD). Chemiluminescent signal was developed by using ECL kit. The western-blotting was quantified by scanning densitometry (Chemi-DOC, Bio-Rad, United States).

### Analysis of Plasma Metabolite by High-Resolution Mass Spectrometry

The plasma samples were homogenized in ultrapure water containing 50% methanol at a ratio of 1:5 (w/v), and 20 μl of the homogenate was added to 180 μl of precipitator containing an internal standard (methanol: acetonitrile = 1:1), mixed by vortexing for 60 s, and centrifuged at 10,000 g for 10 min. Then 5 μl of the sample was used for analysis using a QE-Orbitrap high-resolution mass spectrometer. This detailed procedure was the same as in the literature (Ji et al., 2020).

### Immunoprecipitation Assay

A total of 200 μg of lysates was incubated with 2 μg/ml anti–acetyl-lysine antibodies (1:1,000, Abcam) overnight at 4°C, and then 1/4 of volume protein A/G-agarose beads was added to each sample and incubated on a rotator for 2 h at 4°C as previously described (Fukushima et al., 2016). After 2 h, samples were washed and centrifuged at 15,000 g for 5 min. The prepared samples were detected by western-blotting. The extent of acetylation protein was then subjected to immunoblot analysis as the ratio of acetylated protein/total protein band intensities.

### ATP and Activity of ATP Enzyme Measurement

ATP level and activity of ATP enzyme measurement kits were obtained from Jiancheng Biological Technical Institute (China). In brief, this testing procedure follows as the kits instruct.

### Assay Activity of Metabolic Enzyme

In brief, protein was extracted from atrial tissue or cells lysate with adding protease inhibitors, it was centrifugated at 13,000 rpm/20 min; and then supernatant was taken after centrifugation. IP antibodies were added into supernatant 500ul: LCAD, GDH and AceCS2 antibodies were 1.5 ul, respectively, and incubated overnight in a shaker at 4°C. Reactive protein A-Agarose was taken and washed 3 times with PBS. After washing, about 50% suspension was prepared with PBS. 50 ul of 50% protein A-Agarose suspension was added to the prepared sample and incubated for 2 h on A shaker at 4°C.The supernatant was centrifuged at 4°Cfor 1,500 g for 3 min after incubation. The protein A-Agarose-antibody-protein complex was washed 3 times with pre-cooled PBS. 250 ul ×PBS was added to above complex for resolution testing.

LCAD activity was measured according to the method described (Bharathi et al., 2013). Preparing for reaction compound:100 mM hydroxylamine, 50 mM TrisHCl, 20 mM potassium acetate, 10 mM MgCl_2_, 10 mM ATP, 2 mM DTT, 1 mM ETF-FITC and 1 mM CoA. 20 ul LCAD-IP product and 100 ul double-steam water were added to the bottom hole of the sample, and then 20ul LCAD-IP product and 100 ul reaction compound was added into the sample reaction hole. The fluorescence value was read for 5 min after reaction under 35°C.

GDH activity was detected as follows: preparing for reaction compound (50 mM HEPES, pH 7.5,100 mM NaCl, 1 mM NADH, and 200 μm iodonitrotetrazolium chloride), which was preheated in water bath at 25°C. 180ul reaction compound was added with 20 ul GDH-IP product, and the fluorescence values were determined at 20 s (background value) and 5 min 20 s (sample reaction value). At the same time, the ultra-pure water fluorescence value was taken as the background value.

AceCS2 activity was detected as follows: preparation of reaction compound (100 mM hydroxylamine, 50 mM Tris-HCl, 20 mM potassium acetate, 10mM MgCl_2_, 10 mM ATP, 2 mM DTT, 1 mM ETF-FITC and 1 mM CoA). 20ul AceCS2-IP product and 100ul double-steam water were added to the bottom hole of the sample, and then 20 ul AceCS2-IP product and 100 ul reaction compound were added into the sample reaction hole. The fluorescence value was read for 5 min after reaction under 35°C.

### Immunohistochemical Analysis

The immunohistochemical analysis was determined with the procedures as described previously (Ma et al., 2020). Atria (including appendage and free wall) tissues were fixed in 10% formalin, and then processed for paraffin sections and rehydrated first in xylene and ethanol solutions. The sections were incubated with anti-Sirt3 (1:500; Cell Signaling Technology, United States) overnight at 4°C. The tissue sections were then reacted with peroxidase conjugated rabbit anti-goat IgG (1:1,000, Zhongshan, Beijing, China) at 37°C. Periodic acid Schiff (PAS) staining kit (BA-4044A, Baso, Taiwan) was used to analyze the glycogen distribution in myocytes. There was applied to evaluate the expression of target proteins by the digital image analysis system (HPISA-1000, Olympus, Japan). Positive cell area density was defined as positive cell area/total area of statistical fields.

### Statistical Analysis

All data are represented as mean ± SEM. Statistical significance between different groups was determined by an unpaired *t*-test or a one-way analysis of variance (ANOVA) with the Tukey-test to compare all pairs of columns. When *p* values were less than 0.05, the difference was considered statistically significant.

## Results

### Characteristics of Patients

There were no statistically significant differences in age, weight, body mass index, EF% and medication history between SR group and PAF group (see Table 1).

### Changes of Sirt3 and Glycogen in AF Patients

**FIGURE 1:**
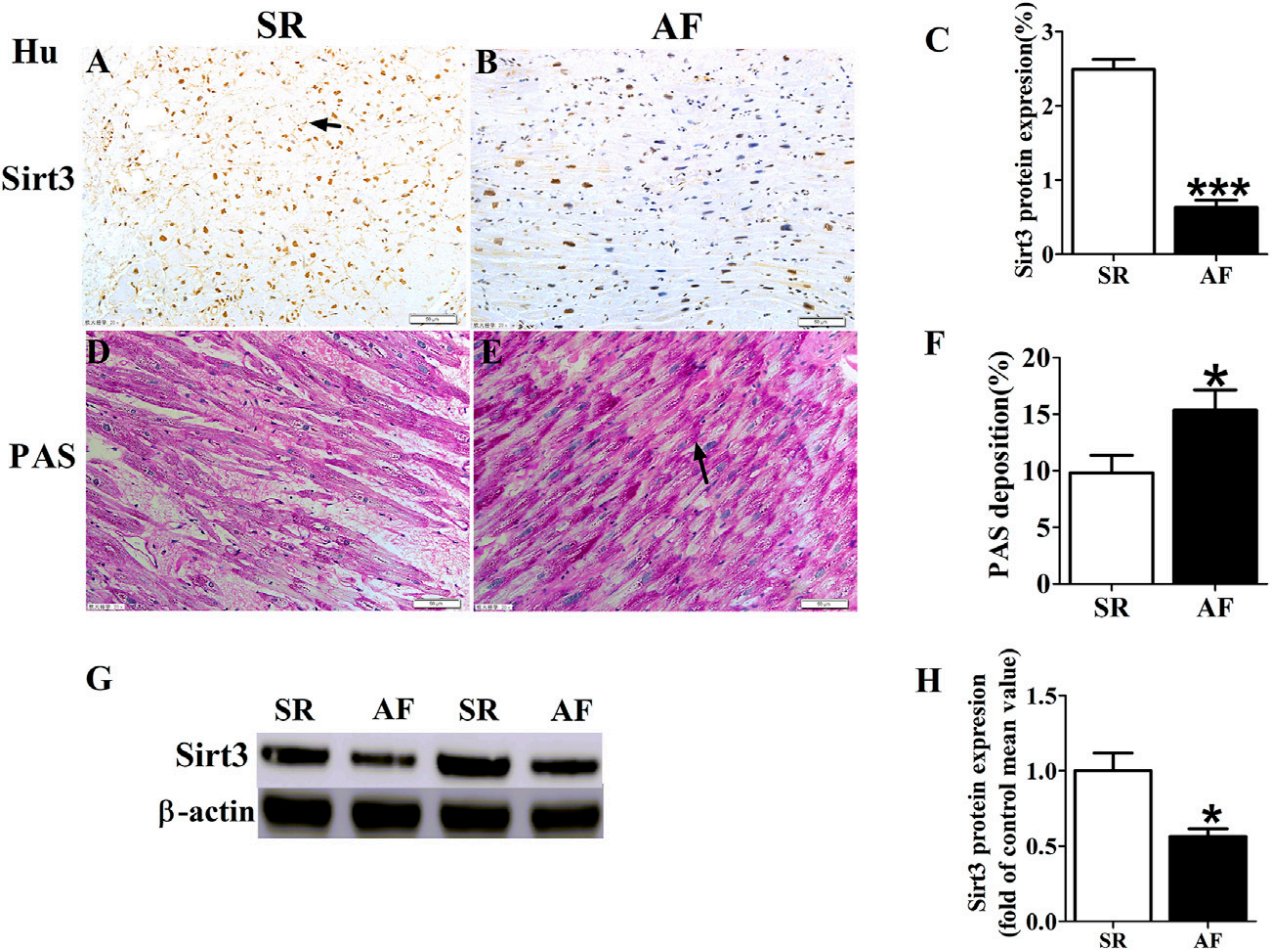
Changes of Sirt3 and glycogen in AF patients. **(A,B and C)** Representative images and statistical results for protein expression of Sirt3 in the atria of patients. **(D,E and F)** Representative images and statistical results for the accumulation of glycogen in both groups of patients. **(G and H)** Representative bands and statistical analyzing for protein expression of Sirt3 in patiets. The magnification is ×20. * *p* < 0.05 vs. SR group, Data from these proteins were normalized to β-actin, n = 6 each group.

We detected the protein expression of Sirt3 in left atrial appendages from SR and AF patients (Figure 1). The immunohistochemical determination and western-blot found that the expression of Sirt3 protein in the AF patients was significantly down-regulated (Figures 1A–C; Figures G,H). Abnormal glycogen accumulation is a remarkable feature of atrial metabolic disturbance. PAS staining showed that AF induced an accumulation of glycogen in the atria, as shown in Figures 1D–F. A large number of red granules were observed in the atrial tissue.

### Electrophysiology Detection and Assay of Glycogen and ATP

**FIGURE 2:**
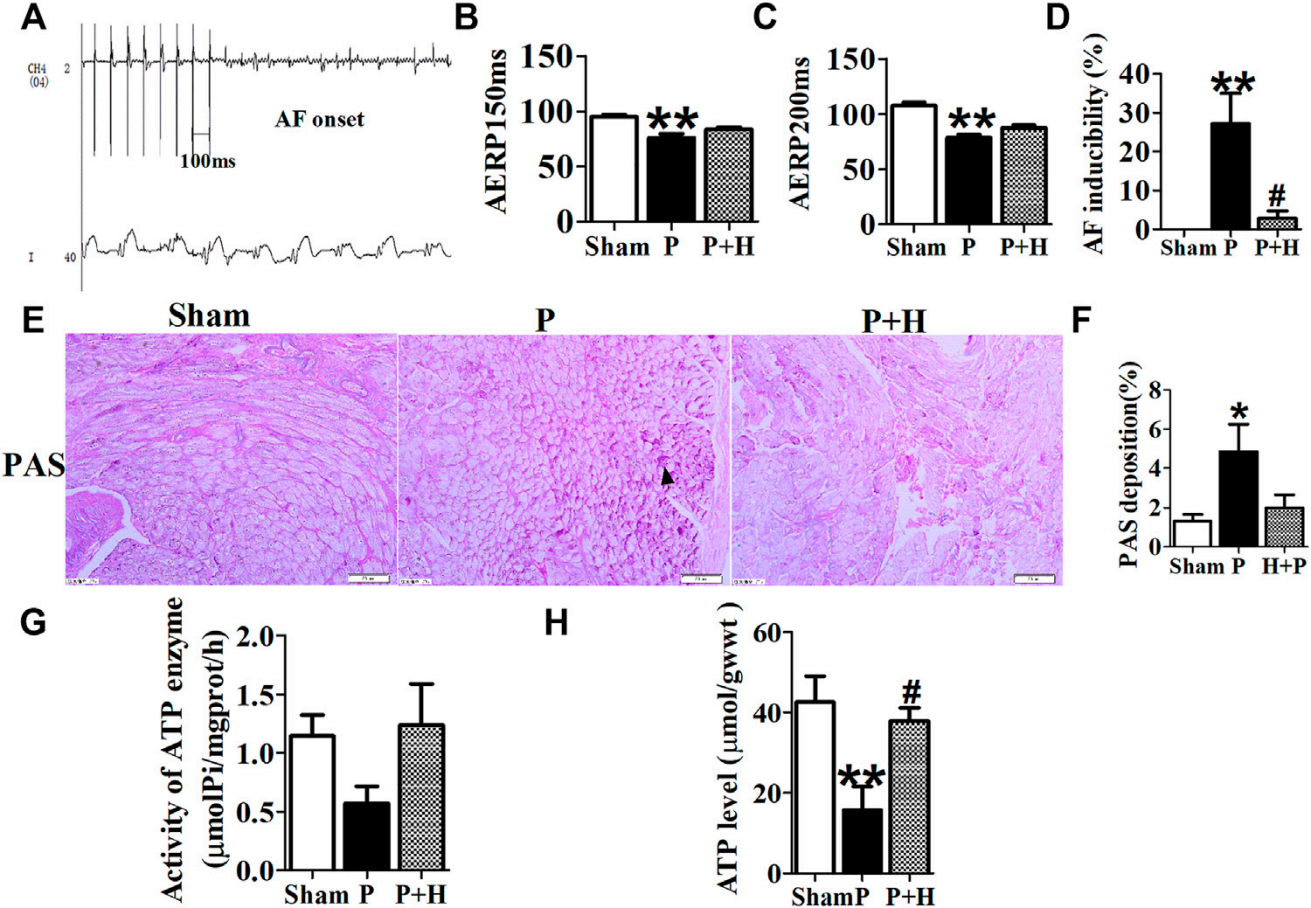
Electrophysiology detection and assay of glycogen and ATP. **(A)** AF was induced after rapid-pacing. **(B,C)** AERP150 ms and AERP200 ms, **(D)** AF inducibility. n = 6 each group. **(E and F)** Representative images and statistical results of the glycogen accumulation in the atria of rabbits, the magnification is ×20. **(G and H)** Statistical analyzes of activity in ATP enzyme and level of ATP. *p < 0.05 vs. sham group,**p < 0.01 vs. sham group, #*p* < 0.05 vs. P group, n = 6 each group.

The results of electrophysiology detection as Figure 2 showed that the AERP_150_ (Figure 2B) and AERP_200_ (Figure 2C) were significantly decreased in the pacing group of rabbits with compared with the sham group, but HL treatment partially inhibited the shorting of AERP by rapid-pacing the atria of rabbits. The AF inducibility was markedly increased in the pacing group compared with the sham rabbits at baseline, but the treatment of Honokiol reversed it (Figure 2D). PAS staining showed that rapid-pacing induced an increase in the glycogen accumulation in the atria, which was partially abrogated by HL (Figures 2E–H). The rapid-pacing induced a decreased in the activity of ATP enzyme, but HL partly inhibited the reduction of ATP enzyme activity, although the activity of ATP enzyme among the three group was not different statistically (Figure 2G). The level of ATP in the pacing group was significantly decreased compared with sham group, however, HL inhibited this change (Figure 2H).

### HL Reversed the AF-Induced Alterations in Circulating Biochemical Metabolites

As shown in Table 3, circulating metabolites were identified and data were processed as described in previous studies (Xie et al., 2018). The metabonomics analysis illustrated that the sham, pacing (P) and HL treatment groups (H + P) could be completely separated (Supplementary Figure S1). Model quality parameters were Accuracy = 0.8, R2 = 0.62, Q2 = 0.89, 53 plasma metabolites in the atria had VIP >1 involved in fatty acid metabolism, glucose and amino acid metabolism, 18 metabolites were increased and 35 of which were decreased in sham/P group, indicating rapid-pacing induced a decline of 18 circulating metabolites and a rising trend of 35 metabolites in the pacing group. Similarly, 17 showed a rising trend and 36 showed a downward trend in H + P/P group, suggesting 17 metabolites were decreased and 36 were increased in pacing group, but HL reversed the changes. MetaboAnalyst4.0 software was used to analyze the main differential endogenous metabolites with VIP value > 1, and the main metabolic pathway with impact value greater than 0.1 was selected. The hearts of Sirt3-deficient mice exhibited more than a 50% reduction in basal ATP content (Ahn et al., 2008), and led to impaired fatty acid oxidation which was correlated with hyperacetylation of LCAD (Hirschey et al., 2010). KEGG analysis was performed on all 53 metabolites with significant differences, and the results showed that fatty acid metabolic pathways, indicating Sirt3, may regulate fatty acid metabolism activated by HL.

**TABLE 3:** The average changes of metabolites (VIP>1) in plasma contributing to discrimination between P group and H + P or Sham group in PLS-DA models (Mean ± SD).

Metabolites	VIP value	H + P/P	Sham/P
LysoPC(18:1 (11Z))	8.7538	109.87 ± 33.22↑	197.17 ± 67.06↑
SM(d18:0/16:1 (9Z))	4.8505	54.61 ± 12.07	66.04 ± 7.89
(7S,8S)-DiHODE	4.5799	1.45 ± 0.95	13.21 ± 29.05
PC(16:1 (9Z)/20:3 (8Z,11Z,14Z))	3.8339	71.35 ± 4.49	70.49 ± 24.6
PC(18:0/20:4 (8Z,11Z,14Z,17Z))	3.5755	79.71 ± 16.44	72.6 ± 13.99
9Z,12Z,15Z)-(7S,8S)-Dihydroxyoctadeca-9,12,15-trienoic acid	3.4277	2.26 ± 1.9	16.36 ± 38.53
SM(d18:1/16:0)	3.2129	54.72 ± 12.42	65.97 ± 8.35
L-Leucine	2.9912	71.56 ± 29.07	42.1 ± 16.1
SM(d18:1/22:0)	2.3943	12,414.9 ± 7,455.65↑	29,165.99 ± 3,567.52↑
L-Acetylcarnitine	2.2603	64.79 ± 51.66	45.15 ± 28.72
LysoPC(16:1 (9Z))	2.1503	120.16 ± 48.19↑	194.67 ± 87.07↑
PC(14:0/22:1 (13Z))	2.1434	158.9 ± 45.95↑	131.21 ± 27.05↑
Phytosphingosine	1.9513	39.7 ± 17.08	73.56 ± 19.06
SM(d18:0/24:1 (15Z))	1.9409	238,784.35 ± 16,798.01↑	224,709.69 ± 51,002.03↑
LysoPC(20:3 (5Z,8Z,11Z))	1.9336	124.21 ± 44.74↑	234.11 ± 81.87↑
Valerylcarnitine	1.8434	21.37 ± 41.91	4.55 ± 3.63
PC(16:1 (9Z)/22:5 (7Z,10Z,13Z,16Z,19Z))	1.8362	96.12 ± 11.15	67.12 ± 22.14
L-Carnitine	1.8091	107.89 ± 66.08↑	154.27 ± 122.87↑
PC(18:1 (9Z)/20:4 (5Z,8Z,11Z,14Z))	1.808	88.54 ± 4.69	80.66 ± 16.74
Linoleic acid	1.6941	79.54 ± 52.93	58.38 ± 66.65
2-Hydroxybutanoic acid	1.6393	78.68 ± 23.79	54.8 ± 22.73
Linoleyl carnitine	1.597	25.31 ± 32.4	13.39 ± 11.23
LysoPC(20:2 (11Z,14Z))	1.579	130.59 ± 40.76↑	229.5 ± 90.19↑
Hypoxanthine	1.5743	81.07 ± 121.41	332.17 ± 485.44↑
(9Z)-(7S,8S)-Dihydroxyoctadecenoic acid	1.5368	4.24 ± 1.78	9.27 ± 16.36
Hexadecasphinganine	1.5018	104.14 ± 29.52↑	80.52 ± 73.25
L-Valine	1.4966	76.8 ± 28.95	85.28 ± 31.63
Propionylcarnitine	1.4793	31.05 ± 59.64	13.22 ± 13.6
Chenodeoxycholic acid	1.4612	46.59 ± 80.13	31.65 ± 29.27
L-Proline	1.4518	83.38 ± 39.72	155.89 ± 22↑
PC(18:2 (9Z,12Z)/P-18:1 (9Z))	1.4426	70.52 ± 12.35	51.71 ± 10.66
11Z-Octadecenylcarnitine	1.3778	29.63 ± 19.96	25.66 ± 13.56
Lactic acid	1.328	76.2 ± 89.3	93.93 ± 91.29
isocitric acid	1.2934	106.34 ± 54.33↑	155.03 ± 39.4↑
SM(d18:1/20:0)	1.2732	395.62 ± 392.34↑	853.05 ± 512.89↑
Arachidonic acid	1.2628	76 ± 43.85	39.16 ± 33.48
Niacinamide	1.249	90.59 ± 64.88	48.99 ± 13.67
PC(14:0/22:0)	1.2306	176.7 ± 79.76↑	146.02 ± 32.71↑
SM(d18:1/18:1 (11Z))	1.1903	41.67 ± 9.75	60.37 ± 17.48
Sphingosine 1-phosphate	1.175	135.9 ± 36.87↑	228.98 ± 127.85↑
PC(o-16:1 (9Z)/18:0)	1.1643	203.38 ± 75.24↑	176.82 ± 91↑
SM(d18:0/18:1 (11Z))	1.1307	77.2 ± 18.12	69.39 ± 24.83
SM(d18:1/24:0)	1.1032	11,122.94 ± 656.4↑	8,706.75 ± 2,919.36↑
LysoPC(14:0)	1.1012	101.44 ± 60.3↑	244.67 ± 184.72↑
PC(o-18:1 (9Z)/20:4 (8Z,11Z,14Z,17Z))	1.0912	79.45 ± 8.38	63.32 ± 14.59
succinic acid	1.0772	64.08 ± 115.03	17.72 ± 15.59
SM(d18:1/22:1 (13Z))	1.0645	200.5 ± 70.03↑	207.66 ± 59.26↑
PC(14:0/22:5 (4Z,7Z,10Z,13Z,16Z))	1.0426	76.11 ± 15.64	82.2 ± 26.1
PC(o-16:1 (9Z)/18:2 (9Z,12Z))	1.0415	64.27 ± 11.47	56.95 ± 5.5
Uric acid	1.036	77.53 ± 93.78	15.96 ± 10.2
Vaccenic acid	1.0322	74.45 ± 46.36	74.33 ± 102.24
Sphinganine	1.0199	64.08 ± 12.87	80.79 ± 36.63

Arrow indicates significantly up-regulated, but no arrow down-regulated metabolites in the H + P and sham groups compared with P group, each group n = 6.

### HL Inhibited the Remodeling of Metabolic Factors and Acetylation Level of Key Metabolic Enzyme in Rabbit of AF Model

**FIGURE 3:**
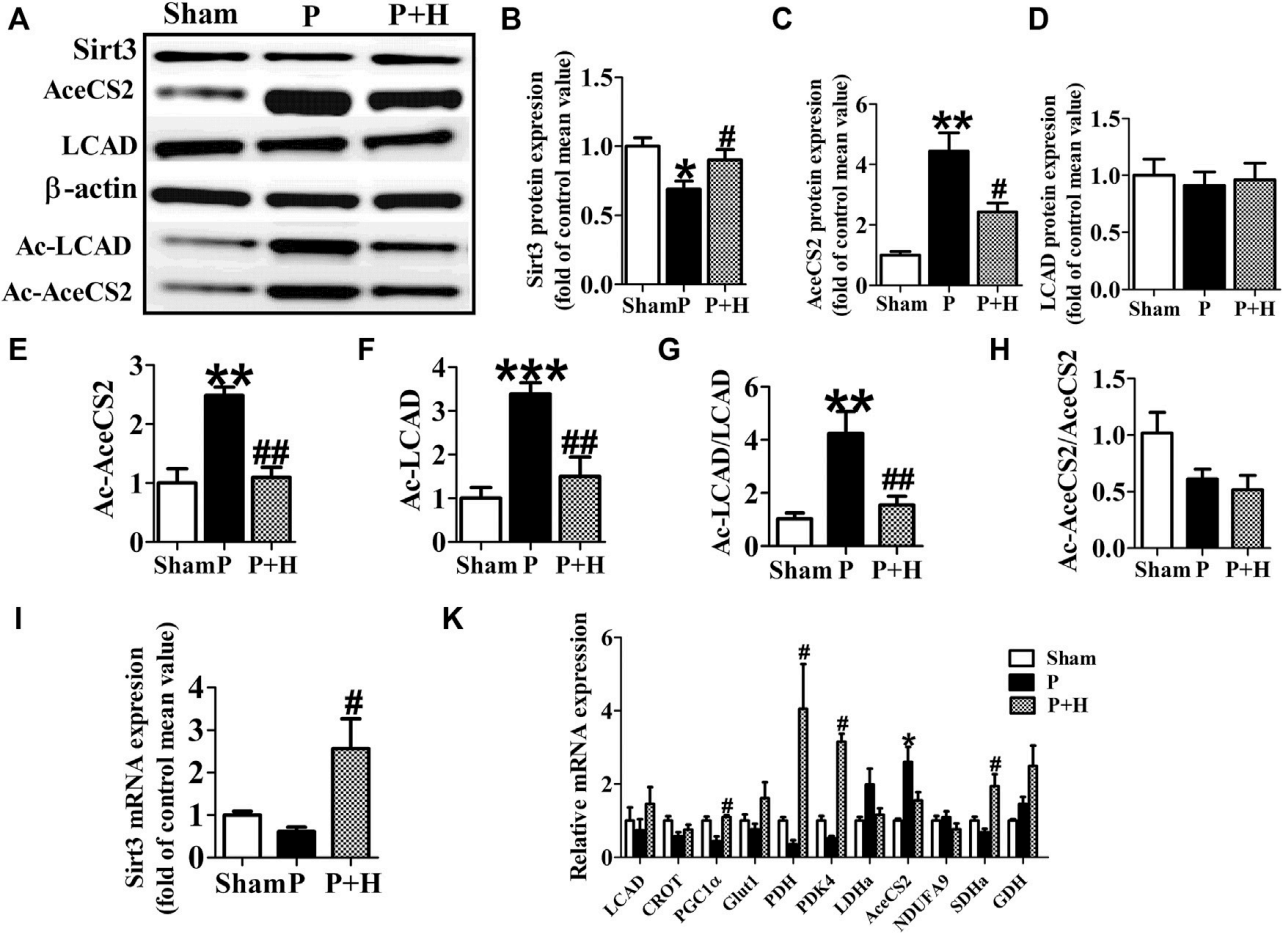
HL inhibited the remodelling of metabolic factors and acetylation level of key metabolic enzyme in rabbit of AF model. **(A)** Representative bands of protein expression of Sirt3, AceCS2, Ac-LACD and Ac-AceCS2. **(B,C,D,E,F)** Quantification analyzing of protein expression of Sirt3, AceCS2, Ac-LACD and Ac-AceCS2. **(G and H)** Statistical results for ratio of Ac-LCAD/LCAD and Ac-AceCS2/AceCS2. **(I)** Statistical results for expression of Sirt3 gene.**(K)** Statistical results for expression of LCAD, CROT, PGC1α,PDH,PDK4, LDHa, AceCS2, NDUFA9, SDHa and GDH genes. Data from these proteins were normalized to β-actin, and genes were normalized to β-actin. *p < 0.05 vs. sham group, **p < 0.01 vs. sham group, ***p < 0.001 vs. sham group; #*p* < 0.05 vs. P group, ##*p* < 0.01 vs. P group, n = 6 each group.

The atria in rabbits that were exposed to rapid pacing significantly decreased the protein and gene expression levels of Sirt3, which was restored by HL (Figures 3A,B,I). Because key metabolic enzymes are acetylated and acetylation can directly affect the enzyme activity or stability, we detected the acetylation level of acetyl-CoA synthetase 2 (AceCS2), a key enzyme involved in tricarboxylic acid cycle metabolism, and of LCAD in fatty acid oxidation. In our study, rapid-pacing induced up-regulated expression of AceCS2 protein (Figures 3A–C), but expression of LCAD protein was not statistically different among all groups of rabbits (Figures 3A–D). However, the acetylation level of AceCS2 and LCAD protein obviously were up-regulated in the pacing group compared with the sham group of rabbits, which was reversed by HL (Figures 3A,E,F). The increased ratio of acetylated AceCS2/AceCS2 protein was found in the pacing group, but HL inhibited this change (Figure 3G).

Next, we detected the mRNA level of metabolic factors including LCAD, CROT, PGC1α, Glut1, PDH, PDK4, LDHa, AceCS2, NDUFA9, SDHa, GDH. Rapid pacing significantly inhibited the expression of LCAD and enhanced AceCS2 mRNA expression; slightly down-regulated CROT, PGC1α, Glut1 and PDH gene expression, and up-regulated LDHa, AceCS2, NDUFA9, SDHa, GDH in atria of pacing group. These changes in expression were restored by HL (Figure 3H).

**FIGURE 4:**
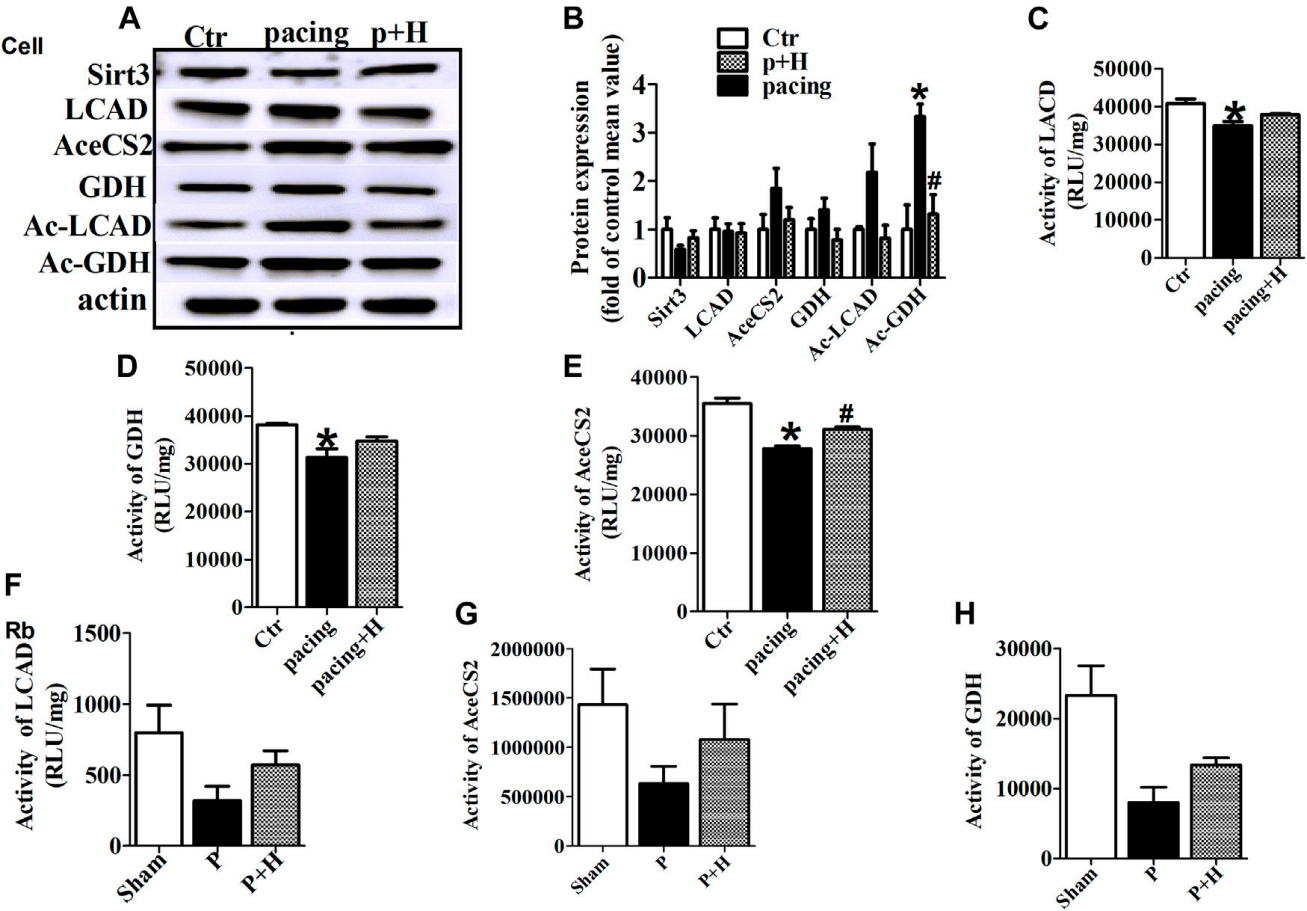
HL inhibited abnormal expression and acetylation of key metabolic enzyme and restored the activity of these enzymes during AF. **(A and B)** Representative bands and statistical results of Sirt3, LCAD, AceCS2, GDH, Ac-LCAD, Ac-GDH in pacing-HL-1 cells. Data from these proteins were normalized to β-actin. **(C,D and E)** Statistical results for activity of LCAD, GDH and AceCS2 in pacing-HL-1cells. *p < 0.05 vs. sham group, #*p* < 0.05 vs. pacing group, n = 3 each group. **(F,G and H)** Statiscal results for activity of LCAD, GDH and AceCS2 in all groups of rabbits. n = 5 each group.

Similarly, HL-1 cells were pretreated with HL (20μM, Sigma-Aldrich, St. Louis, MO, United States) for 1 h (Supplementary Table S1) and then stimulated with rapid pacing for 24 h. The Sirt3 expression levels was decreased, and the acetylation level of LCAD and GDH were increased in the rapid pacing HL-1 cells, but HL inhibited these changes (Figure 4).

### HL Restored the Activity of Key Metabolic Enzyme During Rapid-Pacing Atria

Acetylation is a novel regulatory mechanism for mitochondrial metabolism and controls the activity of key metabolic enzymes. We assessed that rapid-pacing induced a decrease in the activity of LCAD, AceCS2 and GDH *in vivo* and vitro model of AF, and HL administration inhibited the reduction of activity in these metabolic enzymes (Figures 4C,D–F).

### HL Inhibited AF-Induced Metabolic Remodeling *via* Sirt3 Dependent Manner

**FIGURE 5:**
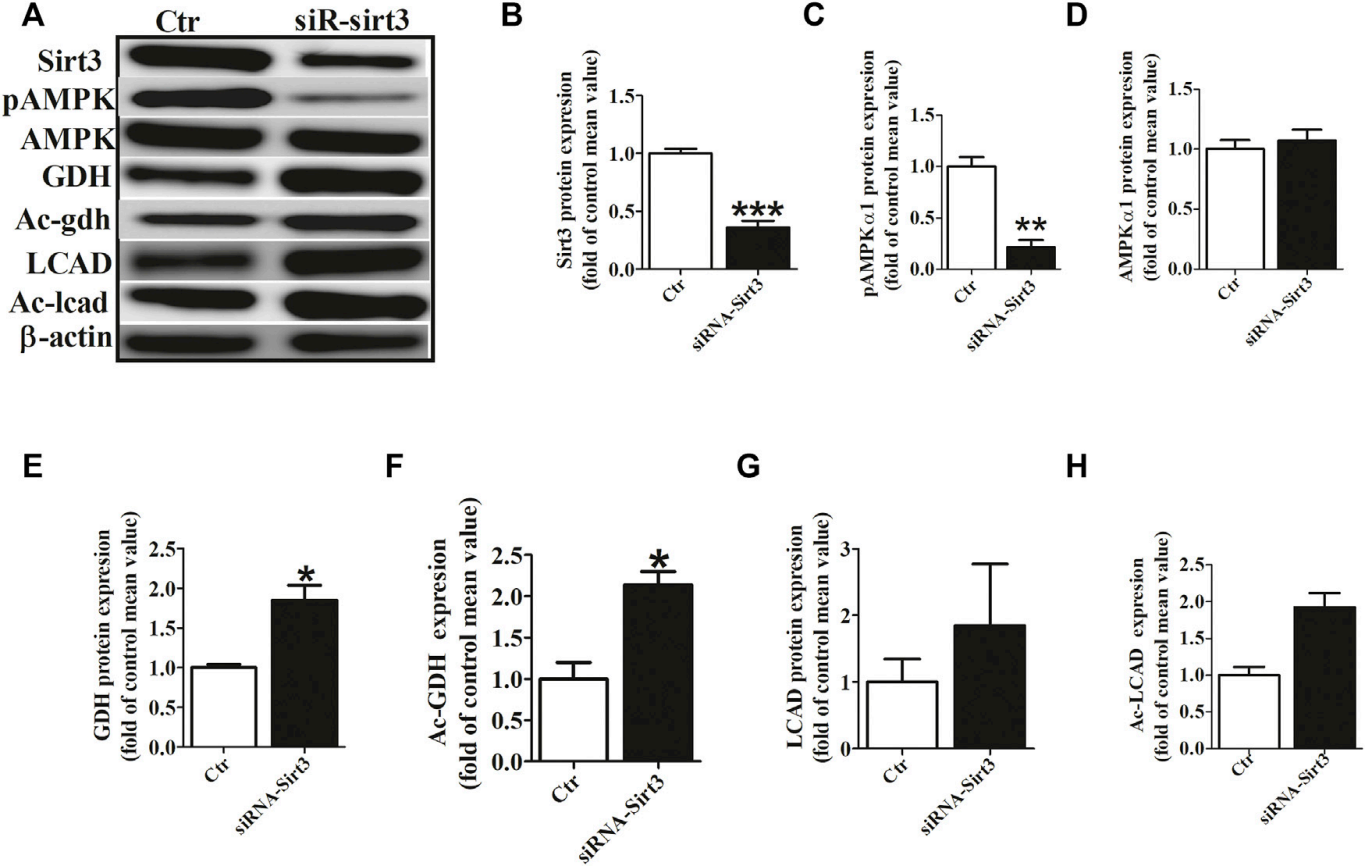
Sirt3 siRNA up-regulated the acetylation level of metabolic enzymes. **(A)** Representative bands of Sirt3, pho-AMPKα1, AMPKα1, GDH, Ac-GDH (Ac-gdh), LCAD and Ac-LCAD expression. **(B)** Statistical results for Sirt3 expression. **(C,D)** Statistical results for pho-AMPKα1and AMPKα1 expression. **(E,F)** Statistical results for GDH and Ac-GDH expression. **(G,H)** Statistical results for LCAD and Ac-LCAD expression. *p < 0.05 vs. control group, **p < 0.05 vs. control group, ***p < 0.05 vs. control group, n = 3 each group.

To investigate the role of Sirt3 in regulating the acetylation of key metabolic enzymes in atria of AF, we knocked down Sirt3 expression with siRNA in HL-l cells. Our data showed that Sirt3 siRNA markedly down-regulated the Sirt3 protein expression after transfection for 48 h (Figures 5A,B), which led to an increase of the mitochondrial acetylation level of GDH and LCAD (Figures 5A,F,G), and a significant decrease of pho-AMPKα1 expression and an increase GDH expression (Figures 5A,C,E). The above results demonstrate that Sirt3 is a key metabolic regulator that regulates the level of acetylation in metabolic enzymes and improves metabolic capacity.

To further determine whether the inhibitory effect of HL on atrial metabolic remodeling during AF was dependent on Sirt3 activation, HL-1cells transfected with the Sirt3 plasmid was used in our study. The HL-1 cells were pretreated with transfection of Sirt3 plasmid for 48 h followed by pacing stimulation or without for 24 h. The expression of Sirt3 was significantly decreased in the pacing group compared to the control group, but the transfection with Sirt3 plasmid abrogated the down-regulation of Sirt3 induced by pacing.

**FIGURE 6:**
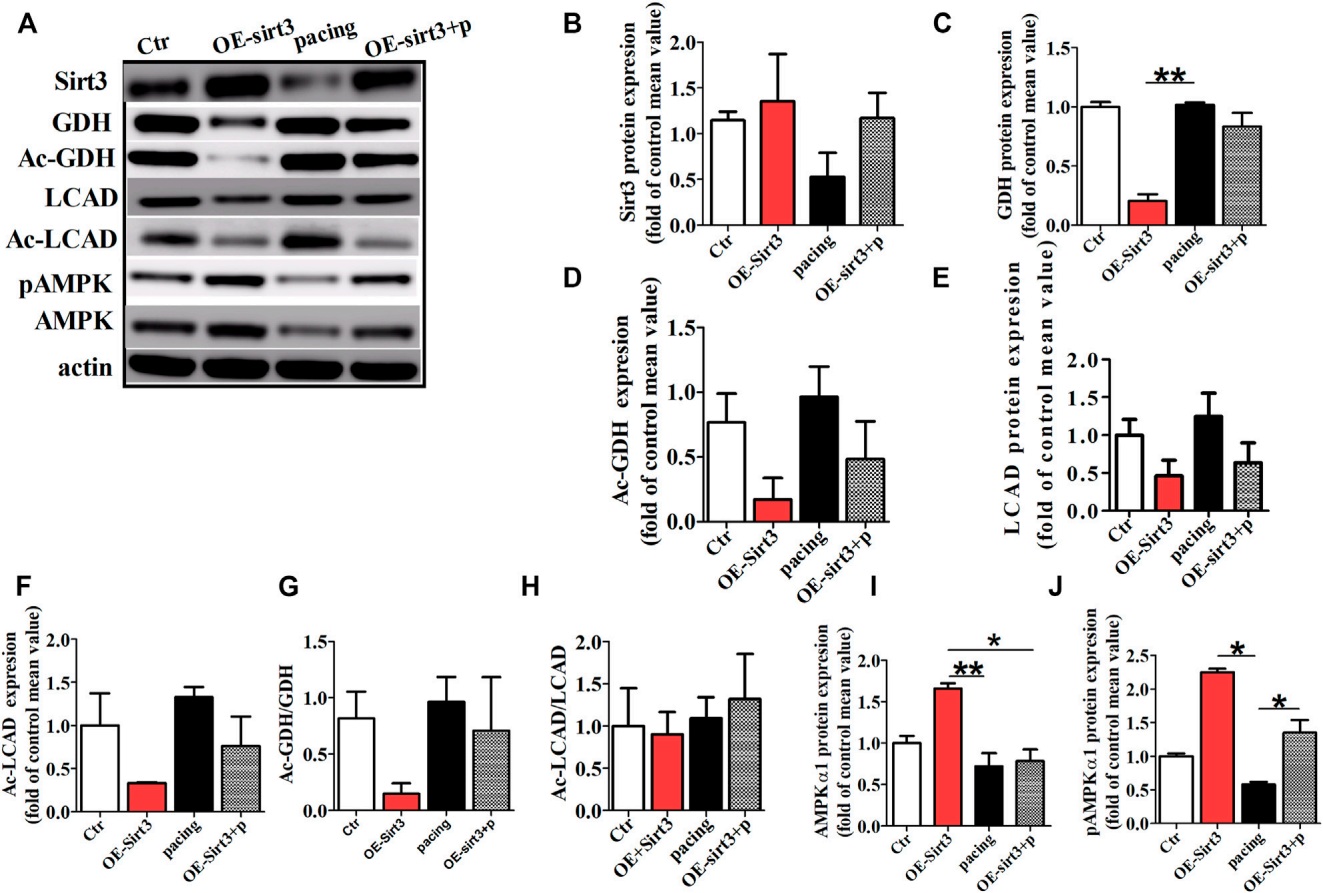
The acetylation level of key metabolic enzymes controlled by Sirt3 pathway *in vitro*. **(A)** Representative bands for the protein expression of Sirt3, GDH, Ac-GDH, LCAD, Ac-LCAD, pAMPK, AMPK in the HL-1 cells of all groups. **(B,C,E,I,J)** Statistical analysis of Sirt3, GDH, LCAD, pAMPK1α and AMPK1α expression. **(D,F,G,H)** Statistical analysis of Ac-GDH, Ac-LCAD, Ac-GDH/GDH, Ac-LCAD/LCAD. *p < 0.05, **p < 0.01, n = 3 each group.

Finally, we assessed the acetylation level of key metabolic enzymes controlled by the Sirt3 pathway *in vitro*. Figure 6 shows that the level of GDH and LCAD expression were down-regulated in the Sirt3 plasmid group, but there was no significant difference among the three groups (Figures 6A,C,E). However, the acetylation level of LCAD and GDH were up-regulated in the pacing group compared with the control group (Figures 6A,D,F,G), which was reversed by Sirt3 plasmid. Similarly, rapid-pacing induced the down-regulation of pAMPKα1, but transfection with Sirt3 plasmid inhibited this change. The above results indicate that HL inhibits the atrial metabolic remodeling of AF *via* a Sirt3-dependent pathway.

## Discussion

This study identified that the down-regulation of Sirt3 in AF patients and the vivo and vitro of the AF model. We found that the increase in acetylation of enzymes involved in mitochondrial fatty acid β-oxidation, glucose oxidation and amino acid metabolism in atria of AF, which led to abnormal changes of metabolites, glycogen deposit, reducing ATP levels, shortening of AERP, and increasing of AF vulnerability. HL reversed the increasing acetylation levels of LCAD, AceCS2 and GDH in animal and cells model of AF. The acetylation modification of key metabolic enzymes was controlled by the overexpression or depletion of Sirt3. Collectively, HL prevented the atrial metabolic remodeling of AF through the Sirt3 dependent pathway.

### HL Reversed AF-Induced Abnormal Atrial Metabolic Remodeling

Our and previous studies found glycogen accumulation and adenine nucleotide reduction in both animal models and humans with AF (Ausma et al., 2000; Liu et al., 2016). HL increased ATP production and reduced lipid peroxidation through the improving mitochondrial function in mice of cisplatin-induced renal injury model (Li et al., 2020). We demonstrated that rapid-pacing caused the reduction of ATP enzyme activity, the level of ATP, and the accumulation of glycogen in rabbits of with the AF model. Our study found an increase in the circulating lactate and ketone body levels in rabbits subjected to rapid-pacing for 1 week (Liu et al., 2016). Similarly, by using metabonomics analysis, we demonstrated that rapid-pacing induced the discordant metabolic alterations of circulating metabolites, which included fatty acid metabolism, glucose oxidation and amino acid metabolism. The results suggest that AF induced metabolic disorder was reversed by HL.

The results in both studies showed the significant down-regulation of transcripts and proteins involved in fatty acid oxidation (Barth et al., 2005; Tu et al., 2014b). Lipid metabolic related gene PGC1α and very-long chain acyl-CoA dehydrogenase (VLCAD) was downregulated in human persistent AF and chronic AF animal models (Bai et al., 2019; Liu et al., 2020). Similarly, we found that rapid-pacing induced a mild decrease of transcript level in LCAD and CROT in atria, indicating AF led to the decrease of fatty acid metabolism, resulting in a reduced level of ATP. As shown in a study and our previous study, the reports suggest that the expression of LDH increased and the expression of Glut4 and PDH decreased in the animal model of AF (Liu et al., 2016; Liu et al., 2020). Consistent with these findings, we found down-regulation of Glut1 and PDH, NDUFA9 and SDH gene, and up-regulation of protein and gene expression in AceCS2 and GDH in both *in vitro* and *in vivo* models of AF, but HL inhibited these alterations. We found that HL treatment partially inhibited the shorting of AERP and the inducibility of AF, and although there is no statistical difference in AERP, it may be related to individual differences in animals.

The above results suggest AF induced dysfunction of glucose transportation and TCA cycle metabolism, increasing glycolysis pathway in atria.

### HL Inhibited Acetylation Modification of Metabolic Enzymes Induced by AF

Post-transcriptional acetylation modification is a potential “regulating valve” of cardiac energy metabolism in AF (Tu et al., 2014a). A previous study found HDAC (Histone deacetylase) inhibitor attenuates atrial remodeling and delays the onset of AF in mice (Scholz et al., 2019). AceCS2 is a key enzyme involved in tricarboxylic acid cycle (TCA) metabolism (Yamamoto et al., 2004), and LCAD is a key enzyme in fatty acid oxidation. Both studies demonstrated that mammalian AceCSS and LCAD were regulated by acetylation and that sirtuins activate AceCS2 and LCAD by deacetylation (Hallows et al., 2006; Hirschey et al., 2010). Moreover, Sirt3 deacetylated GDH and increased its activity (Kim et al., 2012). A study found that cardiac metabolic proteins were hyperacetylated in mice with high-fat diets, which was associated with a decrease in Sirt3 expression (Alrob et al., 2014). Our investigation demonstrated that rapid-pacing induced the increase of acetylation levels in LCAD, AceCS2 and GDH, and led to a decrease in the enzyme activity of LCAD, AceCS2 and GDH during AF, indicating AF induced a decrease in the metabolic capability of fatty acid oxidation, TCA cycle and amino acid metabolism, which were reversed by HL.

### HL Prevented the Atrial Metabolic Remodeling of AF Through Sirt3 Dependent Pathway

Sirt3 plays a critical role in regulating the acetylation of key metabolic enzymes (He et al., 2019). LCAD is deacetylated in wild-type mice under fasting conditions and by Sirt3 *in vitro* and *in vivo*, and LCAD is hyperacetylated in the absence of Sirt3^7^. AceCS2 is abundant in heart and skeletal muscle and it plays an important role in acetate conversion for energy production. AceCS2 is a highly conserved and metabolic enzyme from bacteria to human, catalyzes the conversion of acetate to acetyl-CoA, and enables peripheral tissues to utilize acetate during fasting conditions (Shimazu et al., 2010). The acetyl-CoA is an important substrate for the tricarboxylic acid cycle. Accumulating biochemical studies have linked Sirt3 with activation of the mitochondrial enzyme AceCS2 that Sirt3 can regulate acetylated-modification of AceCS2 (Hallows et al., 2006; Schwer et al., 2006). In our study, we demonstrated AF induced an increase of acetylated LCAD and AceCS2 protein levels, following the reduction of its enzyme activity, indicating a decrease of metabolic capacity in the fatty acid β-oxidation and tricarboxylic acid cycle during AF. Furthermore, GDH is an amino-acid metabolic enzyme that promotes the metabolism of glutamate and glutamine, resulting in the generation of ATP, which promotes insulin secretion. There is a similar effect of acetylated-GDH level and its enzyme activity, which is regulated by Sirt3(Kim et al., 2012). Consistent with our results, AF increased acetylation level of GDH, following a decrease of its activity, and indicating AF induced the decrease of amino-acid metabolism in atria.

The above study was consistent with our research, and our results demonstrate that overexpression or depletion of Sirt3 could regulate the acetylated-modification of LCAD, AceCS2 and GDH enzymes *in vitro* model of AF, in contrast change of activity in key metabolic enzymes. Further, increasing Sirt3 could improve metabolic capacity by regulating the acetylation level in metabolic enzymes. The above results indicated Sirt3 is a key metabolic regulator.

Sirt3 agonist HL could ameliorate cardiac hypertrophy by activating Sirt3 (Pillai et al., 2015) and improving fatty acid oxidation resulting in the inhibition of acute kidney injury induced by cisplatin (Li et al., 2020). However, the effects of HL on AF are not known. Our present study found a significant down-regulation of Sirt3 expression in rabbits, cell models of AF, and AF patients, followed by a reduction in LCAD, AceCS2 and GDH activity, and the acetylation level of its expression, following a decrease of fatty acid metabolism, TCA and amino-acid metabolism, but HL reversed the above changes. Interestingly, these small changes in the expression of metabolic factors in AF rabbits induced the derangement of atrial fatty acid metabolism, glucose and amino acid metabolism, which was consistent with the findings of previous studies (Liu et al., 2016), (Liu et al., 2020). In this study, HL prevented the atrial metabolic remodeling of AF through the Sirt3 dependent pathway.

Sirt3 has been implicated in various cardiac pathologies and it deacetylates multiple enzymes in mitochondrial metabolism (Yamamoto et al., 2004). A previous study reported that Sirt3 and AMPK stimulated mitochondrial biogenesis, which increased mitochondrial turnover and cardiomyocyte regeneration (Xin and Lu, 2020). In our study, the increase or decrease of Sirt3 could regulate the expression of pho-AMPKα1, suggesting Sirt3 is a controller of AMPK. In the present study, HL could up-regulate the expression of Sirt3, resulting in improving atrial metabolic remodeling and inhibiting AF. These findings indicated that HL inhibited metabolic remodeling of AF *via* regulating the Sirt3 signaling pathway.

## Conclusion

We demonstrated that the Sirt3 dependent pathway participated in atrial metabolic remodeling during AF and that HL inhibited atrial metabolic remodeling by regulating the Sirt3 dependent pathway. Thus, this pathway may provide a new potential therapeutic method for AF. Our study provided a novel insight into the pharmacological role of HL against AF and atrial metabolic remodeling.

## Data Availability

The original contributions presented in the study are included in the article/Supplementary Materials, further inquiries can be directed to the corresponding authors.
