# Supplementary material for: Honokiol Inhibits Atrial Metabolic Remodeling in Atrial Fibrillation Through Sirt3 Pathway
Source: Front Pharmacol. 2022 Mar 17;13:813272. doi: 10.3389/fphar.2022.813272 (PMC8970047; doi:10.3389/fphar.2022.813272)
Supplement: Supplementary file 1 [file Table1.docx]

| Sample | OD value | | | | | | Mean | Inhibition |  |
| --- | --- | --- | --- | --- | --- | --- | --- | --- | --- |
| con | 0.669 | 0.680 | 0.761 | 0.660 | 0.723 | 0.787 | 0.713 | 0.00% | 0.713 |
| 40uM Honokiol | 0.858 | 0.821 | 0.742 | 0.779 | 0.820 | 0.766 | 0.798 | -11.82% | 0.713 |
| 20uM Honokiol | 0.814 | 0.904 | 0.790 | 0.697 | 0.785 | 0.846 | 0.806 | -12.99% | 0.713 |
| 10uM Honokiol | 0.683 | 0.716 | 0.605 | 0.639 | 0.810 | 0.744 | 0.700 | 1.94% | 0.713 |
| 5uM Honokiol | 0.641 | 0.706 | 0.713 | 0.679 | 0.766 | 0.692 | 0.700 | 1.94% | 0.713 |
| 2.5uM Honokiol | 0.513 | 0.587 | 0.624 | 0.603 | 0.611 | 0.628 | 0.594 | 16.68% | 0.713 |
|  |  |  |  |  |  |  |  |  |  |
| 20uM Honokiol has a proliferation effect on cells, and the proliferation effect is the highest. Thus, 20uM Honokiol is selected for HL-cell experiments. | | | | | |  |  |  |  |

**TableS1 Effect of different concentration of Honokiol on cell proliferation**
